# Copper metabolism-related Genes in entorhinal cortex for Alzheimer's disease

**DOI:** 10.1038/s41598-023-44656-9

**Published:** 2023-10-14

**Authors:** Yan Zhang, Yu-shen Yang, Cong-mei Wang, Wei-can Chen, Xin-li Chen, Fan Wu, He-fan He

**Affiliations:** 1 Department of Anesthesiology, The Second Affiliated Hospital of Fujian Medical University, No. 34 North Zhongshan Road, Quanzhou, 362000 Fujian Province China; 2 Department of Anesthesiology, Shishi General Hospital, No. 2156 Shijin Road, Shishi, 362700 Fujian Province China

**Keywords:** Computational biology and bioinformatics, Neuroscience, Diseases, Molecular medicine, Neurology

## Abstract

The pathological features of Alzheimer's disease are the formation of amyloid plaques and entanglement of nerve fibers. Studies have shown that Cu may be involved in the formation of amyloid plaques. However, their role has been controversial. The aim of this study was to explore the role of Cu in AD. We applied the “R” software for our differential analysis. Differentially expressed genes were screened using the limma package. Copper metabolism-related genes and the intersection set of differential genes with GSE5281 were searched; functional annotation was performed. The protein–protein interaction network was constructed using several modules to analyse the most significant hub genes. The hub genes were then qualified, and a database was used to screen for small-molecule AD drugs. We identified 87 DEGs. gene ontology analysis focused on homeostatic processes, response to toxic substances, positive regulation of transport, and secretion. The enriched molecular functions are mainly related to copper ion binding, molecular function regulators, protein-containing complex binding, identical protein binding and signalling receptor binding. The KEGG database is mainly involved in central carbon metabolism in various cancers, Parkinson's disease and melanoma. We identified five hub genes, FGF2, B2M, PTPRC, CD44 and SPP1, and identified the corresponding small molecule drugs. Our study identified key genes possibly related to energy metabolism in the pathological mechanism of AD and explored potential targets for AD treatment by establishing interaction networks.

## Introduction

Alzheimer's disease (AD) is a complex neurodegenerative disease that is the result of a combination of factors, characterised by the accumulation of amyloid (Aβ) plaques and neuronal fibrillary tangles of Tau proteins^1–4^. In the 1990s, the “amyloid cascade” hypothesis became the dominant hypothesis^5,6^, with results suggesting that senile plaques are pathogenic^5^. Subsequently, drugs have been developed based on this hypothesis^7^. However, to date, no single causal treatment has achieved the desired effect, possibly because only a single factor has been considered^8–10^. Therefore, it is reasonable to rethink the role of Aβ. In fact, there are conflicting accounts of the role of Aβ deposits. Aβ is not necessarily harmful because in a physiological setting, the formation and accumulation of Aβ fibrils is part of the metabolite Amyloid precursor protein (APP), and it is probably the Aβ oligomers that have toxic effects^11^. Several studies have suggested that amyloid-rich plaques can be conceived as "net traps" where toxic substances and infectious agents are “trapped”^12,13^, thus providing a protective effect on the body. It follows that Aβ, APP, and tau proteins play critical roles in the neural pathways associated with AD. In additional, multiple pathogenic pathways may activate disease cascades through independent mechanisms. For example, Apoe, lipid metabolism and inflammation. Apoe, a risk factor for AD, is mainly involved in lipoprotein metabolism^14^, which maintains brain morphology and homeostasis and plays an important role in the aging process^15^, and Apoe binds to trigger receptor 2 (TREM2) expressed by myeloid cells, which is mediated by microglia in the central nervous system (CNS)^16^. Therefore, microglia are one of the major cells involved in the pathogenesis of AD. Activated microglia promote the production of inflammatory cytokines and chemokines, which increase the activity of β-site APP cleaving enzyme (BACE1) and nuclear factor κB (NFκB), leading to an increase in the production of Aβ^17^, while the aggregation of Aβ provides unlimited stimulation to microglia, which further increases the level of activated microglia^18^. Activated microglia also induce other signaling pathways, such as the PI3K/Akt pathway, which is involved in the regulation of apoptosis and inflammatory responses; activation of PI3K also promotes NFκB translocation^19^. Overall, these cellular pathways all interact and are inextricably linked.

In addition, the relationship between AD and Cu has been reported^20^, and it has been demonstrated that AβPP/Aβ has a high affinity for copper^21^, forming a Cu-Aβ complex. When the Cu-Aβ complex is overloaded, it leads to the production of toxic compounds that cause oxidative stress^22^, contributing to neuronal degeneration and leading to cognitive dysfunction. However, there is considerable controversy regarding the role of Cu in the development of AD, with one meta-analysis showing that brain Cu is deficient in AD patients compared to normal brain tissue specimens^23^. In contrast, most studies have shown high levels of Cu in AD^24–30^.

Copper is involved in several physiological pathways in the body^31^, and in the brain, copper is essential for neuronal function as it is involved in biological processes such as neurotransmitter synthesis and respiratory oxidation^32,33^. A recent study has shown that many different copper-binding molecules or ion carriers can induce cell death by a mechanism that involves the accumulation of intracellular copper, a mode of death that differs from known forms of cell death (e. g. apoptosis, iron death, etc.), and which the research team identified as a new form of cell death and named-Cuprotosis^34^. However, the mechanism of copper ion carrier-induced cytotoxicity is still unknown. Indeed, dysregulation of copper homeostasis has been clearly linked to neurological disorders, where decreases and increases in brain copper (respectively) lead to neurodegeneration, including Wilson and AD. In AD, copper is thought to be the substance that directly causes molecular changes in the brain^35^.

AD is a major medical condition. Due to the complexity of its pathological mechanism, its diagnosis and treatment are uncertain. Copper-induced neuronal death may be a newly discovered pathological process that could prove helpful for understanding the pathogenesis of AD more comprehensively. Therefore, in this study, we attempted to identify the hub gene related to copper metabolism in AD by mining the data obtained by gene chip technology and to find new drug targets for AD treatment.

## Materials and methods

### Data source

Gene expression profile data, including microarray, gene expression, and chip data, were obtained from an open functional genomics high-throughput resource database, the Gene Expression Omnibus (GEO) database (https://www.ncbi.nlm.nih.gov/geo/)^36^. We searched the GEO database for relevant gene expression datasets using the terms 'Alzheimer's disease' (research keyword) and 'human' (organism). Finally, we downloaded the GSE5281 dataset, which contains 161 brain tissue samples, and measured six brain regions. The brain region of interest (entorhinal cortex) was selected for our analysis, which included 8 AD and 13 healthy samples. Table 1 report the demographics of the individuals.

**Table 1:** The demographics of the individuals.

	Con (n = 13)	AD (n = 8)	P value
Age (yr, Mean ± SD)	80.31 ± 9.20	84.13 ± 5.54	0.250
Sex			0.032
Male	10 (76.9%)	2 (25%)	
Female	3 (23.1%)	6 (75%)	

### Differential expression analysis

We applied the “R” software (R v4.2.1) for our differential analysis. First, we converted the probe sets in file formats into gene symbols using human annotation packages. Probe sets without gene symbols were then removed, and the average expression values for probe sets sharing the same gene symbols were retained. The data were normalized using a robust multi-array averaging algorithm. Finally, DEGs were screened using the limma package (R 3.4.3). We set adj. *p* < 0.05, and |logFC (fold change) |> 2 to DEGs. Subsequently, we searched the GeneCards database for genes related to copper metabolism, crossed them with GSE5281, identified DEGs related to copper metabolism, and generated a Venn map of DEG using the online tool JVenn.

### Enrichment analyses of copper-related DEGs

For functional enrichment analysis of DEGs, g: Profiler (http://biit.cs.ut.ee/gprofiler/ gost), Metascape (https://metascape.org/gp/index.html#/main/step1), and WebGestalt (https:// www.webgestalt.org/) were used. These enrichment analysis tools have different algorithms that can verify one another. Gene Ontology (GO) classification comprises molecular function (MF), biological process (BP), cellular component (CC), and Kyoto Encyclopedia of Genes and Genome (KEGG) pathway enrichment analyses. We uploaded DEGs related to copper metabolism to the over-representation analysis (ORA) of WebGestalt for further research. Molecular function (MF), BP, and CC were analyzed separately. In addition, we uploaded the obtained DEGs related to copper metabolism using the online gene function annotation analysis tool, Metascape. The annotation of biological processes was completed using Metascape. The pathway enrichment analysis was mainly obtained from g: Profiler, which included the Reactome database and wikipathways database besides MF, CC, BP, and KEGG.

### Protein–protein interaction (PPI) network construction, module analysis, and identification of hub genes

To further explore the interactions between the genes obtained above, we constructed a PPI network using the STRING database (http://STRING-db.org/) to reveal the general organizing principles of functional cellular systems and to predict protein interactions. In the network results, the nodes represent the protein, and the lines represent the interactions between the proteins. Modular analysis and visualization of PPI network results were performed using molecular complex detection technology (MCODE). Using the default parameters (degree cutoff value 2, node score cutoff value 2, K-core 2, and maximum depth = 100), we identified 10 hub genes using five different algorithms using the CytoHubba plug-in and selected genes that were in the five algorithms for subsequent analysis.

### Small molecule agents screening and molecular-ligand docking analysis

Five common genes were mapped to the corresponding drugs using Network Analyst (https://www.networkanalyst.ca/). Network Analyst is a comprehensive online platform for gene expression analysis and network visualization analysis that can help discover drug-gene interactions in regulatory networks. The protein crystal structure of gene was downloaded from the Protein Data Bank database (http://www.rcsb.org, PDB). We then used AutoDock Tools software (version 1.5.7) to molecularly dock the small molecule compounds with corresponding target. Pymol software (http://www.pymol.org) was used to evaluate the binding activities of small molecule compounds and targets. Furthermore, we conducted an analysis of 6 hub genes for pharmacogenetic interactions utilizing the DGIDB data resource (https://dgidb.genome.wustl.edu/). This resource furnishes information regarding the correlation between genes and established or potential medications.

## Results

### Identification of DEGs

**Figure 1:**
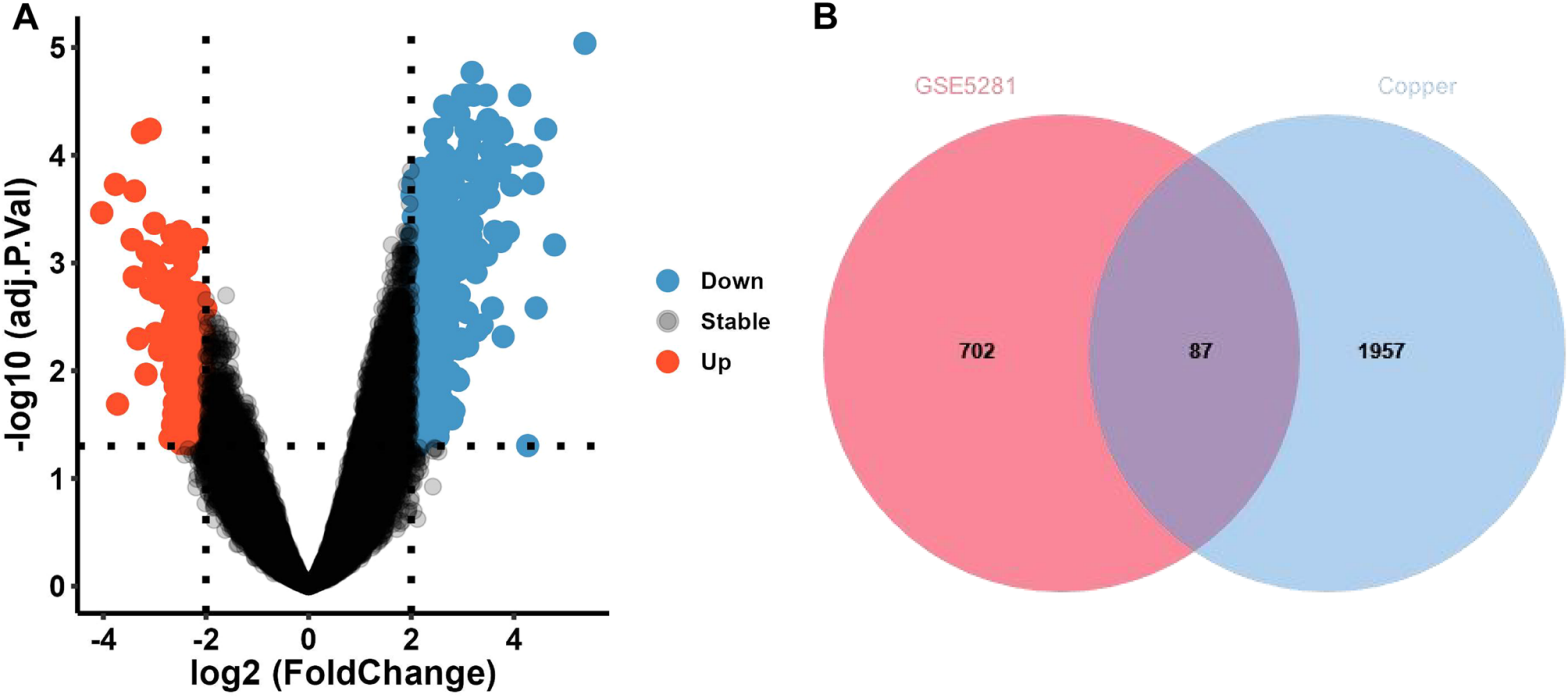
Volcano plot illustrating DEGs. (**A**) The volcano plot shows the DEGs between the control group and AD, red represents up-regulated genes, blue represents down-regulated genes, and gray represents genes with little fold change. (**B**) Venn plots show common genes associated with copper metabolism in GSE5281. DEGs, differentially expressed genes.

**Figure 2:**
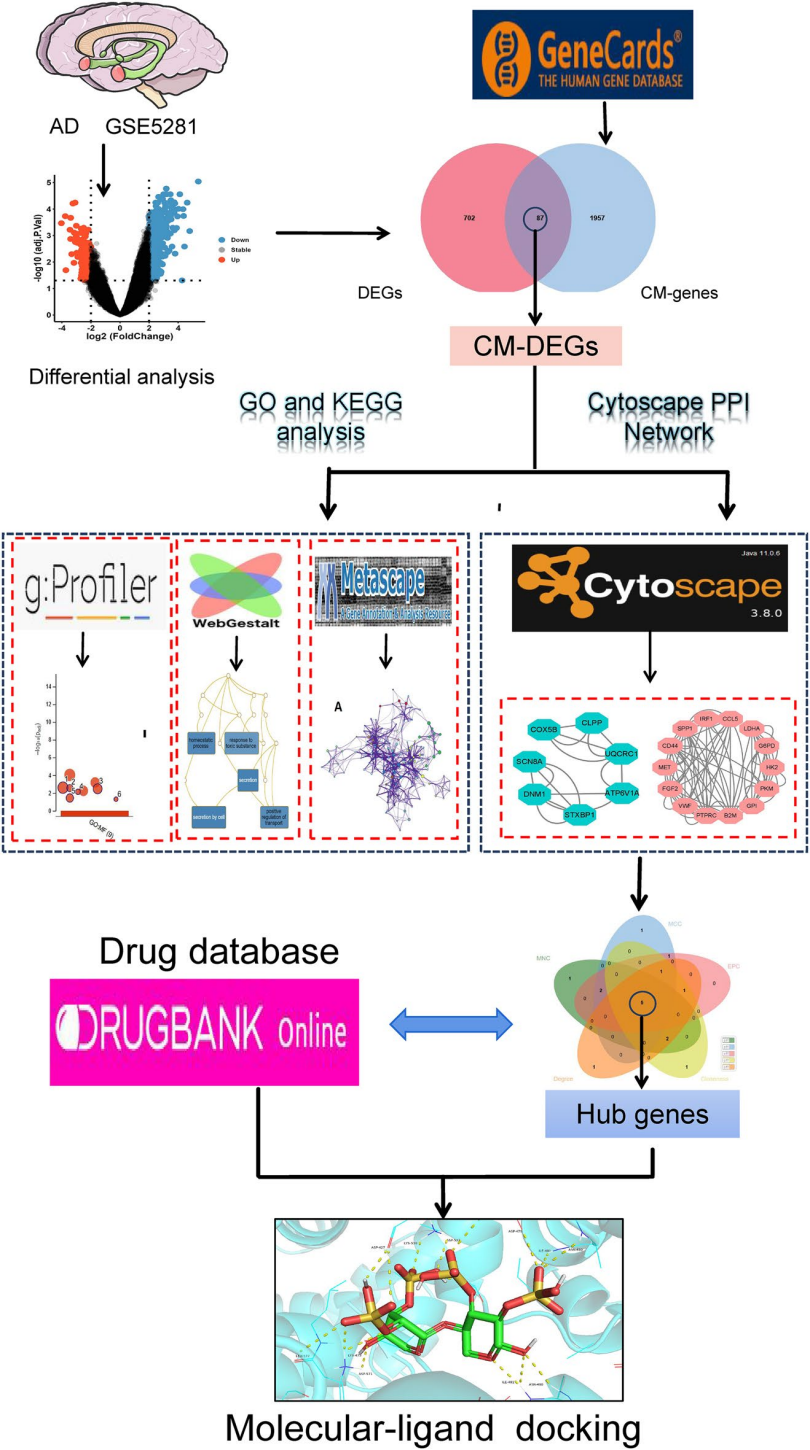
The visual flow-process diagram of this study. AD: Alzheimer's disease, CM-genes: Copper metabolism-related genes, DEGs: Differentially expressed genes, GO: Gene Ontology, KEGG: Kyoto Encyclopedia of Genes and Genome.

Through analysis of the GSE5281 dataset using the “R” software, the differentially expressed genes between patients with AD and normal groups are shown in the volcano plot (Fig. 1A). 231 up-regulated genes and 561 down-regulated genes were obtained. Similarly, we obtained 2044 genes related to copper metabolism from the GeneCards database and cross-identified them with GSE5281. The results are shown in Fig. 1B. In total, 87 CM-DEGs were identified. The information on the entire research process is shown in Fig. 2.

### Analysis of the functional characteristics of common DEGs

**Figure 3:**
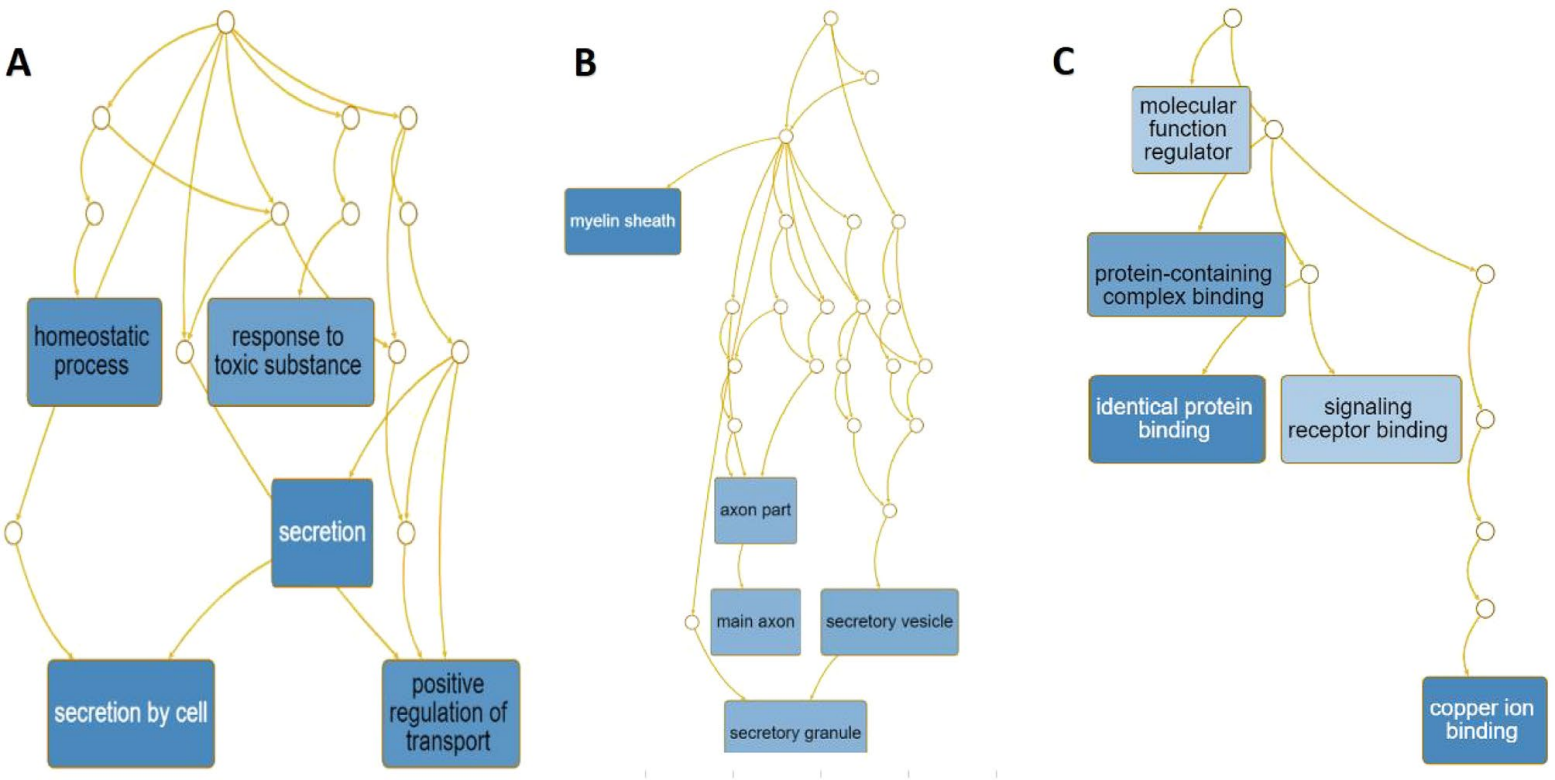
Gene Ontology analysis. Biological process (BP,** A**); cellular component (CC,** B**); molecular function (MF,** C**); and analysis results of 87 DEGs with copper metabolism.

**Figure 4:**
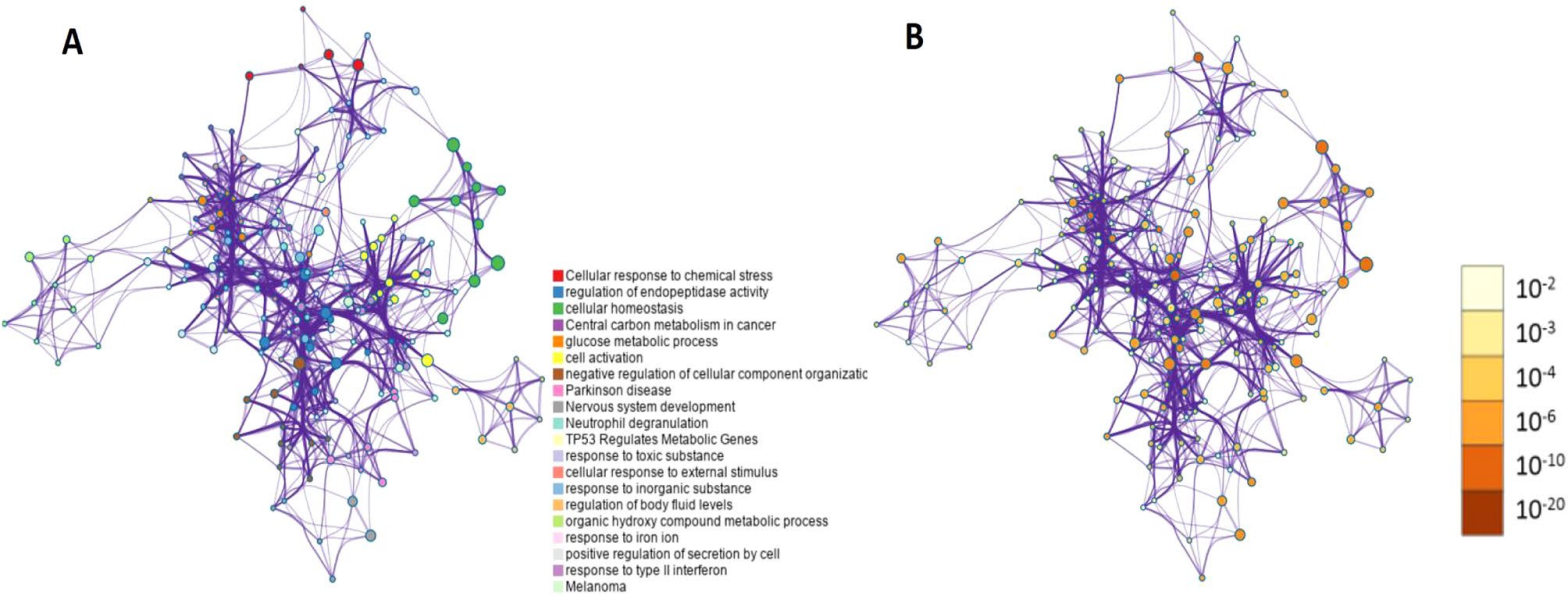
Network of enriched terms. (**A**) colored by cluster ID, where nodes that share the same cluster ID are typically close to one another; (**B**) colored by p-value, where terms containing more genes tend to have a more significant p-value.

**Figure 5:**
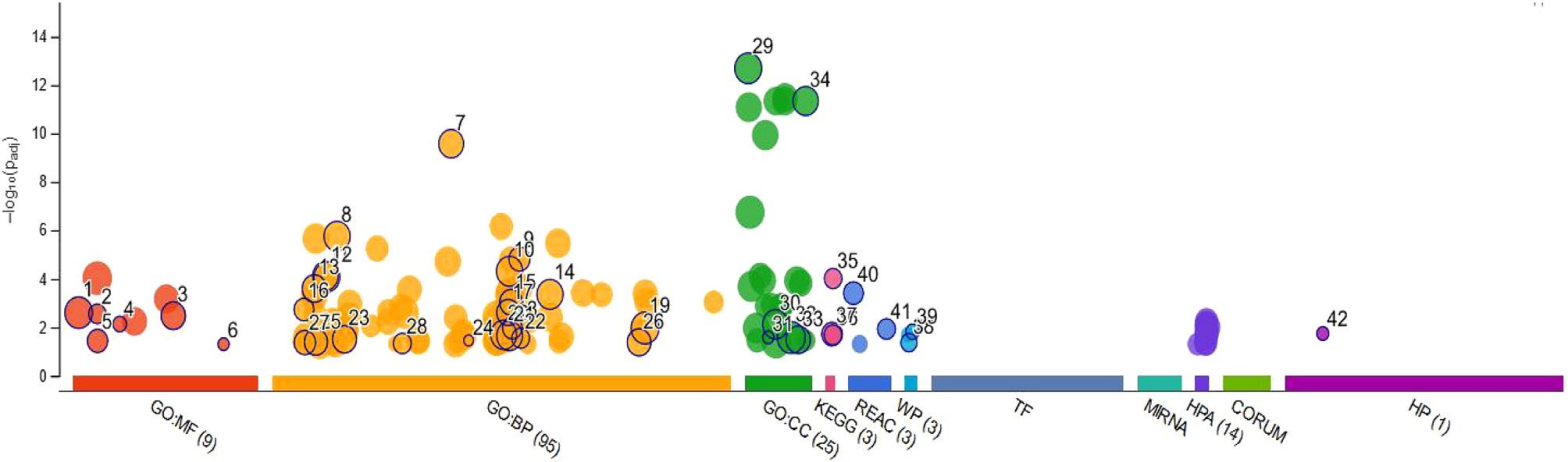
g:Profiler performs functional enrichment analysis, also known as over-representation analysis (ORA) or gene set enrichment analysis, on DEGs list. In addition to Gene Ontology, it includes pathways from KEGG Reactome and WikiPathways. Red represents MF, orange represents BP, green represents CC, pink represents KEGG, blue represents REAC.

To further explore the capabilities of the 87 CM-DEGs, we performed feature and pathway enrichment analysis using g Profiler (http://biit.cs.ut.ee/gprofiler/gost), WebGestal, and Metascape. First, we sent the related information on DEGs to WebGestal for GO analysis. The results of the analysis of the enriched gene datasets are shown in Fig. 3. These genes were mainly enriched in homeostatic process, response to toxic substance, positive regulation of transport, and secretion. The enriched molecular functions were mainly related to copper ion binding, molecular function regulator, protein-containing complex binding, identical protein binding, and signaling receptor binding. Moreover, WebGestal results were further verified using Metascape. The results are shown in Fig. 4, with specific annotations are presented in Table 2. g: Profiler was used to analyze the pathways of 87 CM-DEGs in the samples. The website mainly includes the KEGG, Reactome, and Wikipathways databases. The KEGG database focuses on diseases such as central carbon metabolism in various cancers, Parkinson's and melanoma, while the REAC database is enriched for cellular responses to chemical stress, TP53 regulation of metabolic genes and detoxification of reactive oxygen species. Glycolysis during ageing, aerobic glycolysis and amyotrophic lateral sclerosis are the main pathways in the WP database. (Fig. 5 and Table 3).

**Table 2:** Top 10 GO annotation in metascape.

GO	Category	Description	Count	%	Log10(P)	Log10(q)
R-HSA-9711123	Reactome gene sets	Cellular response to chemical stress	11	12.64	− 10.86	− 6.51
GO:0,052,548	GO biological processes	Regulation of endopeptidase activity	12	13.79	− 9.78	− 5.73
GO:0,019,725	GO biological processes	Cellular homeostasis	15	17.24	− 9.14	− 5.32
hsa05230	KEGG pathway	Central carbon metabolism in cancer	7	8.05	− 8.88	− 5.23
GO:0,006,006	GO biological processes	Glucose metabolic process	8	9.20	− 8.72	− 5.15
GO:0,001,775	GO Biological Processes	Cell activation	14	16.09	− 7.77	− 4.93
GO:0,051,129	GO biological processes	Negative regulation of cellular component organization	14	16.09	− 7.74	-4.93
hsa05012	KEGG pathway	Parkinson disease	9	10.34	− 7.11	− 3.94
R-HSA-9675108	Reactome gene sets	Nervous system development	12	13.79	− 6.99	− 3.87
R-HSA-6798695	Reactome Gene sets	Neutrophil degranulation	11	12.64	− 6.86	− 3.76

**Table 3:** Paths to 3 databases enrichment in g profiler.

Database	Term name	Term ID	Padj
KEGG	Central carbon metabolism in cancer	KEGG:05,230	9.579 × 10^–5^
	Parkinson disease	KEGG:05,012	1.901 × 10^–2^
	Melanoma	KEGG:05,218	2.064 × 10^–2^
REAC	Cellular response to chemical stress	REAC:R-HSA-9711123	3.868 × 10^–4^
	TP53 regulates metabolic genes	REAC:R-HSA-5628897	1.171 × 10^–2^
	Detoxification of reactive oxygen species	REAC:R-HSA-3299685	1.171 × 10^–2^
WP	Glycolysis in senescence	WP:WP5049	1.516 × 10^–2^
	Aerobic glycolysis	WP:WP4629	2.009 × 10^–2^
	Amyotrophic lateral sclerosis (ALS)	WP:WP2447	4.293 × 10^–2^

### PPI network analysis of DEGs associated with copper metabolism

**Figure 6:**
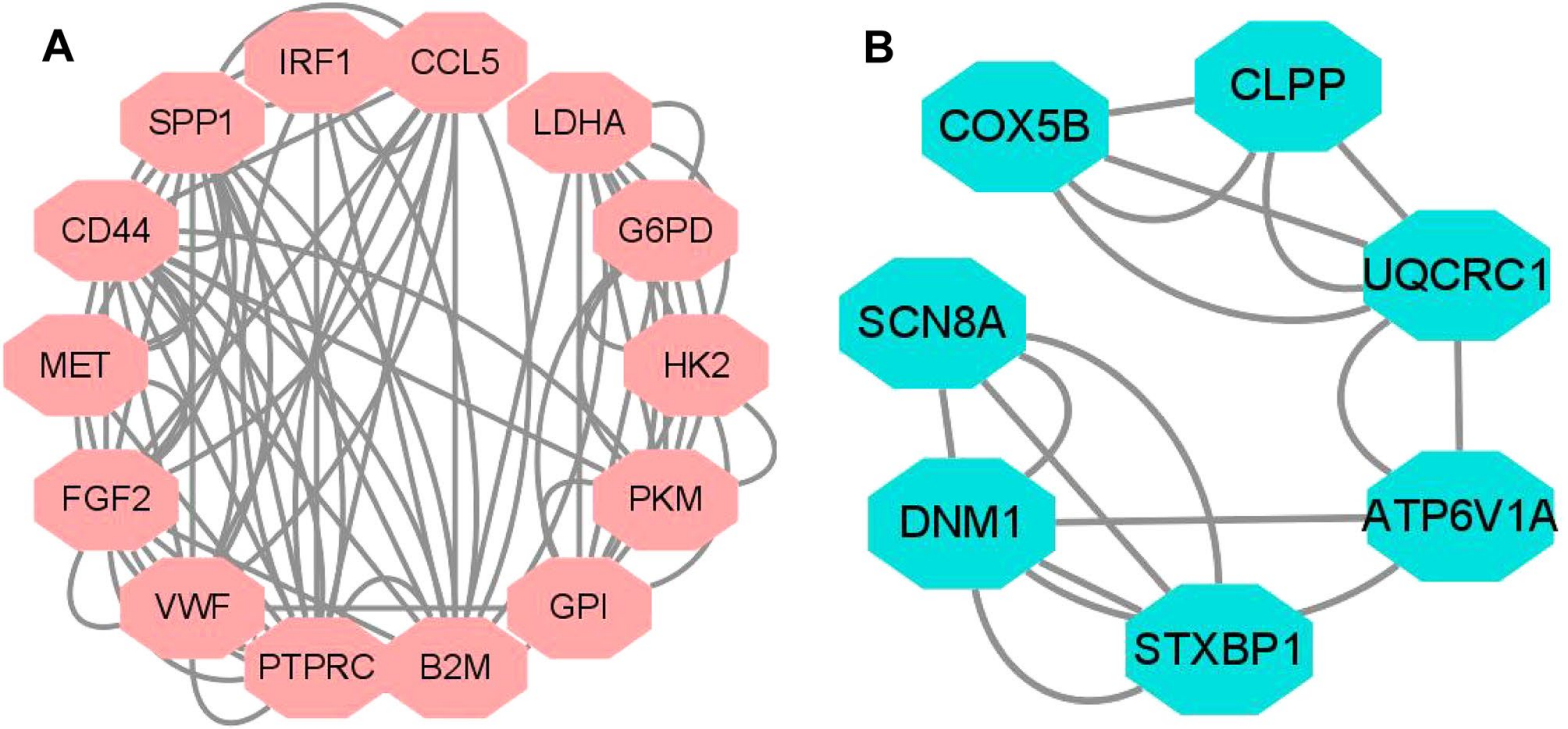
The two most prominent modules in the PPI network. (**A**) is module 1, (**B**) is module 2.

**Figure 7:**
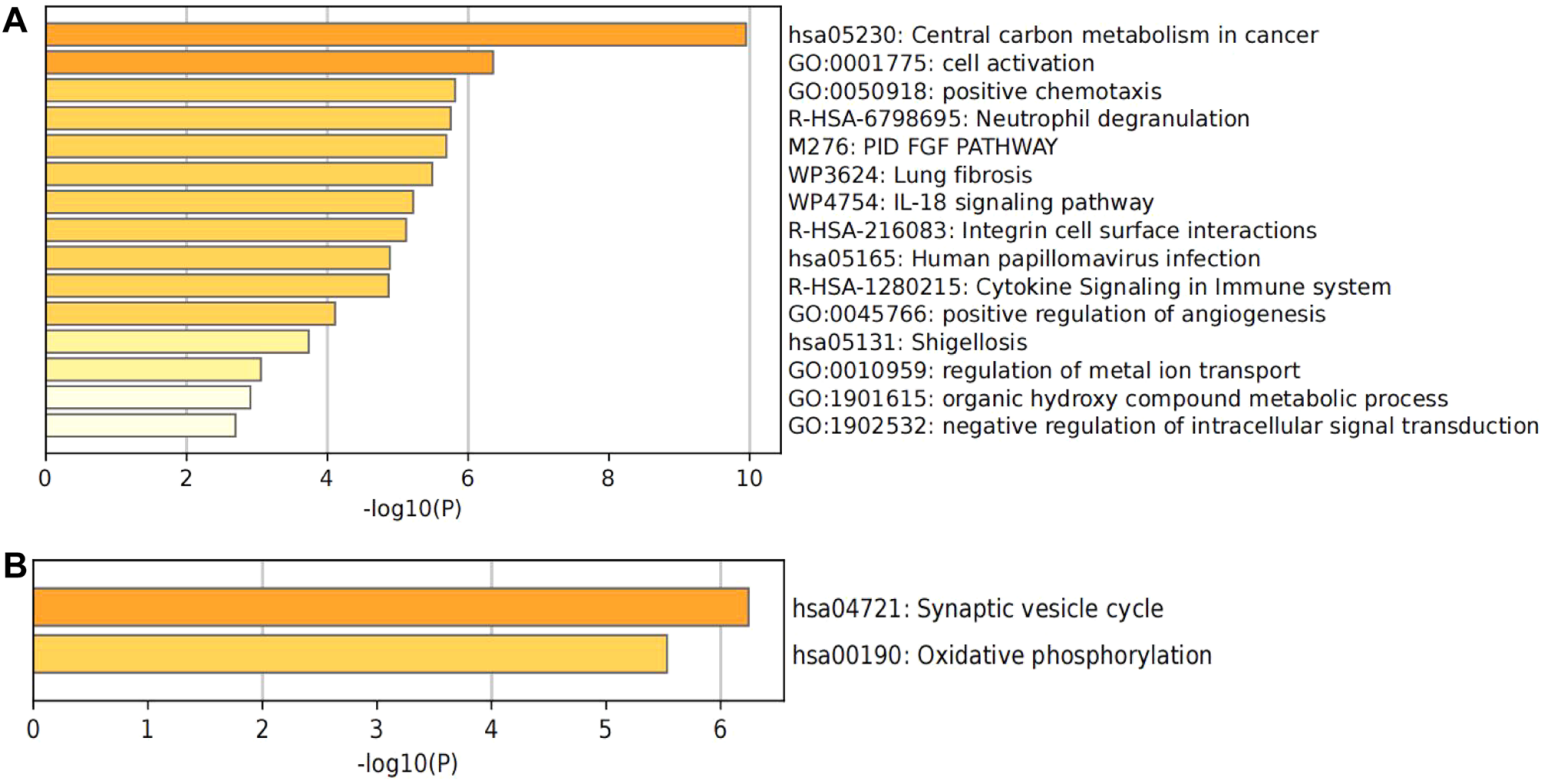
GO analysis of two modules. Bar graph of enriched terms across modules gene lists, colored by *p*-values. (**A**) module 1. (**B**) module 2.

PPI analysis was performed to identify interactions between differentially expressed genes associated with copper metabolism. The results showed an interaction between these genes related to copper metabolism, and we obtained a PPI network consisting of 87 nodes and 171 edges. Using Cytoscape clustering analysis of the gene network with the MCODE software, two key modules were constructed (Fig. 6A,B). Metascape was used to analyze the functions of the two modules. Module 1 mainly focused on central carbon metabolism in cancer, cell activation, and positive chemotaxis. Modules 2 involved relatively few genes, including fsynaptic vesicle cycle and oxidative phosphorylation. (Fig. 7). Additionally, we identified the top 10 hub genes using five algorithms (Table 4) and selected the crossed five genes, fibroblast growth factor 2 (*FGF2*), Beta-2-Microglobulin (*B2M*), and Secreted Phosphoprotein 1 (*SPP1*), CD44, and Protein Tyrosine Phosphatase Receptor Type C (PTPRC) for subsequent analysis.

**Table 4:** CytoHubba's 5 algorithms ranking top 10 genes, and bold type is a common gene.

MNC	MCC	EPC	Degree	Closeness
**FGF2**	**PTPRC**	**B2M**	**FGF2**	**B2M**
**B2M**	**FGF2**	**FGF2**	**B2M**	SNCA
**CD44**	CCL5	**SPP1**	SNCA	**FGF2**
**PTPRC**	**CD44**	**CD44**	**PTPRC**	**CD44**
**SPP1**	**SPP1**	**PTPRC**	**CD44**	**PTPRC**
COL1A1	**B2M**	COL1A1	**SPP1**	**SPP1**
CCL5	VWF	CCL5	PKM	LDHA
GSN	COL1A1	VWF	LDHA	TXN
LDHA	RUNX2	RUNX2	HSP90AB1	HSP90AB1
HSP90AB1	IL7	SNCA	RUNX2	RUNX2

### Drug-gene crosstalk and functional analysis of potential genes

**Figure 8:**
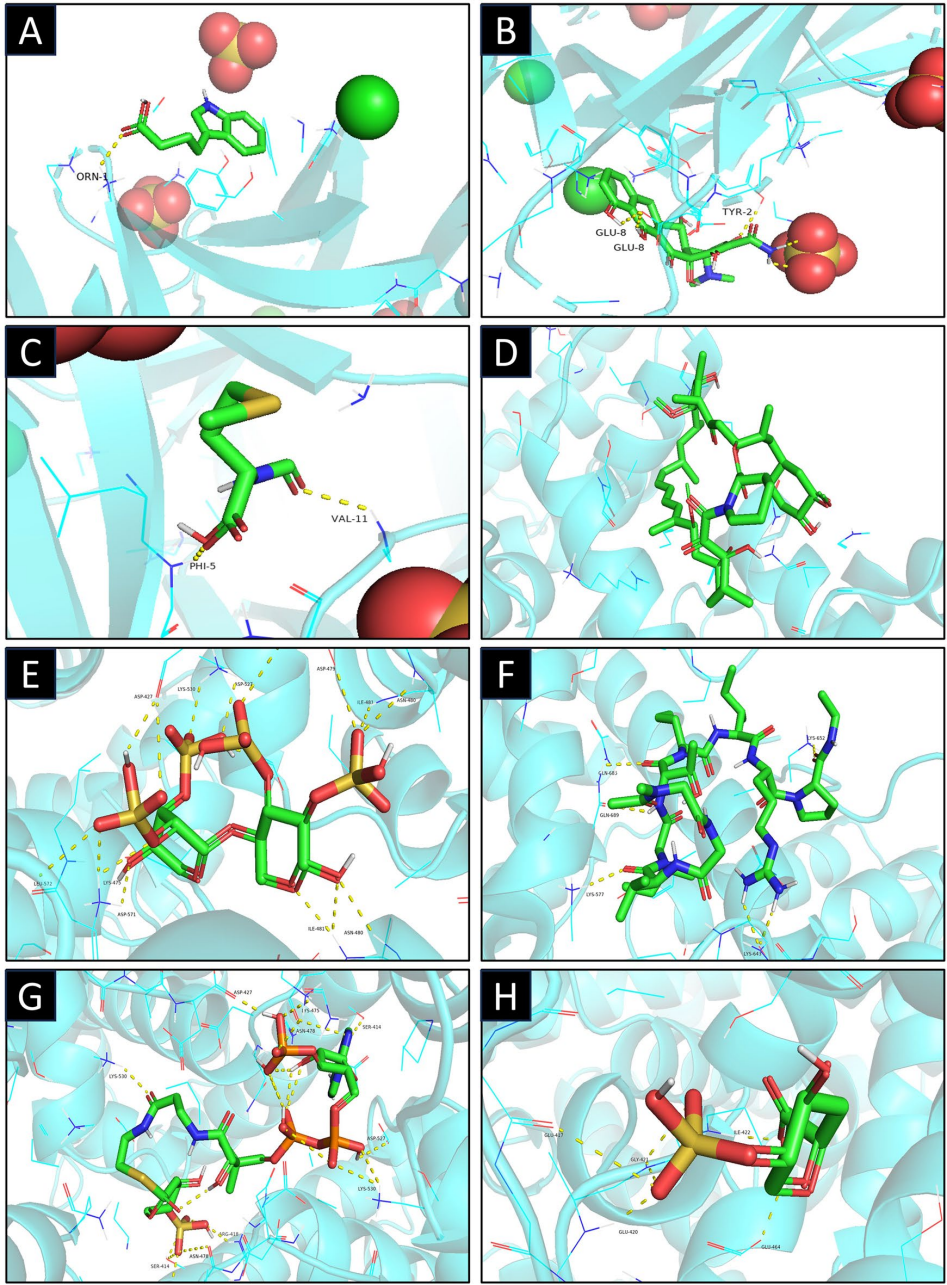
Molecular docking pattern of the compounds identified in the DrugBank database with the corresponding targets (B2M and F2F). (A) B2M-3-indolebutyric acid [affinity (kcal/mol): − 5.4]; (**B**) B2M-doxycycline [affinity (kcal/mol): − 7.3]; (**C**) B2M-n-formylmethionine [affinity (kcal/mol): − 3.8]; (**D**) F2F-sirolimus [affinity (kcal/mol): − 8.2]; (E) F2F-pentosan polysulfate [affinity (kcal/mol): − 6.9]; (**F**) F2F-ABT-510 [affinity (kcal/mol): − 6.4]; (**G**) F2F-1,4-dideoxy-O2-sulfo-glucuronic acid [affinity (kcal/mol): − 8.3]; (**H**) F2F-1,4-dideoxy-5-dehydro-O2-sulfo -glucuronic acid [affinity (kcal/mol): − 6.0].

Uploading these five genes to Network Analyst revealed that only FGF2 and B2M had related compounds in the DrugBank database, which might provide potential therapeutic targets for AD. In order to present and clarify the interaction between the compounds and the corresponding targets, molecular docking was performed. Molecular docking is a useful method to display the optimal conformation of target molecules and small molecule compounds for interaction. In the current study, the crystal structures of two molecular targets [(F2F, PDB ID: Q14209; Resolution: 2.20 Å), and (B2M, PDB ID: P61769; Resolution: 1.91 Å)], were obtained from the RCSB Protein Data Bank. Then AutoDock Tools1.57 software was used to dock compounds and the two molecular targets with the largest fold difference. It’s reported that when the docking scores were less than − 6 kcal/mol, the binding affinity of compounds with the targets was high. Figure 8 showed the binding poses and sites, where the green color represents the compounds, and the yellow dotted lines represent hydrogen bond interactions.

We additionally analyzed the drug-gene interactions of the five hub genes using DGIDB data resources (https://dgidb.genome.wustl.edu/). The results showed that six drugs interacted with the *SPP1* gene, among which GENTAMICIN also interacted with CD44, FGF2, and PTPRC, which were closely related to ten different drugs, and no related drug was found for β2M. Six drugs have been studied for their effects on AD (ADALIMUMAB^37^, ETANERCEPT^38^, INFLIXIMAB^39^, ESTRADIOL^40^, PREDNISONE^41^, PROGESTERONE^42^), while the effect of remaining 22 drugs on AD remains to be revealed (Table 5).

**Table 5:** Candidate drugs targeting genes with AD.

Drug	Gene	Interaction score	Clinical application
SUCRALFATE	FGF2	5.89	Approved
REBAMIPIDE	FGF2	1.96	Approved, investigational
FAMOTIDINE	FGF2	1.47	Approved
ABT-510	FGF2	1.47	Investigational
PYRAZOLE	FGF2	1.47	Experimental
THYROTROPIN	FGF2	1.18	Investigational
SQUALAMINE	FGF2	0.74	Investigational
QUIZARTINIB	FGF2	0.65	Approved, investigational
FP-1039	FGF2	0.59	Unsearchable
PHENYLEPHRINE	FGF2	0.42	Approved
HYDROGEN PEROXIDE	PTPRC	10.3	Approved, vet approved
CHEMBL204543	PTPRC	5.15	Unsearchable
APAMISTAMAB IODINE I-131	PTPRC	5.15	Investigational
ALENDRONIC ACID	PTPRC	1.29	Approved
**ADALIMUMAB**	PTPRC	0.91	Approved, experimental
**ETANERCEPT**	PTPRC	0.81	Approved, investigational
**INFLIXIMAB**	PTPRC	0.77	Approved
EPOETIN BETA	PTPRC	0.74	Approved
**ESTRADIOL**	PTPRC	0.34	Approved, investigational, vet approved
**PREDNISONE**	PTPRC	0.32	Approved, vet approved
HYALURONAN	CD44	6.87	Approved, vet approved
GENTAMICIN	CD44	1.96	Approved, vet approved
**PROGESTERONE**	CD44	0.62	Approved, vet approved
ASK-8007	SPP1	10.3	unsearchable
CALCITONIN	SPP1	3.43	Approved, investigational
GENTAMICIN	SPP1	0.98	Approved, vet approved
ALTEPLASE	SPP1	1.08	Approved, investigational
TACROLIMUS	SPP1	0.61	Approved, investigational
WORTMANNIN	SPP1	0.74	Experimental

The bold text represents drugs that have been studied in relation to Alzheimer's disease.

## Discussion

AD is a refractory neurodegenerative disease that has a detrimental effect on quality of life, especially in older adults^43,44^. The most typical pathological features of AD patients are amyloid plaque deposits and neurofibrillary tangles. In the last four decades, the scientific community has made a great deal of research on β-amyloid (Aβ), from pathological mechanisms to drug development. However, the results have not been satisfactory. Therfore, the scientific community has rethought the amyloid doctrine and the intervention strategies derived from it, while the need for new targets has grown more urgent.

The tremendous advances and widespread applications of bioinformatics prediction and computer technology in recent years have facilitated the exploration of more viable biomarkers and novel therapeutic candidates for various diseases. An increasing amount of sequence data is being submitted to public databases such as cancer genome maps and GEO databases. Through bioinformatics analysis, we identified 789 DEGs between AD and normal samples and then identified DEGs related to copper metabolism. GO and KEGG analyses were performed on these DEGs to explore their potential molecular mechanisms and linkages.

Notably, aerobic glycolysis in the wikipathways database is closely related to mitochondria. Mitochondrial dysfunction can lead to a variety of disorders such as neurodegenerative disorders AD and Parkinson’s disease (PD). The pyruvate dehydrogenase complex (PDHC) and 2-ketoglutarate dehydrogenase complex showed reduced activity in the affected areas of the AD brain^45^. In the AD cerebral cortex, the activities of complex I, complex II-III, and cytochrome oxidase are decreased^46^. AD increases oxidative damage to the mitochondrial DNA^47^. In addition, Lynn and coworkers^48^ analyzed changes in the mitochondrial protein group in temporal pole (TP) brain specimens from patients with mild cognitive impairment, early AD, and late AD and identified a list of 21 proteins with increased expression in patients with mild cognitive impairment. These proteins belong to the electron transport chain tricarboxylic acid pathway, chaperone, and ATP transport and utilization^48^.

Metal ions play a critical role in numerous biological processes and neuronal activity in the brain, including copper, iron, and zinc. Therefore, it is important to regulate metal ion levels in the brain for optimal function and health. Uncontrolled fluctuations of these metal ions in the brain can lead to homeostatic imbalances in the internal environment and result in massive cell death. The concept of Ferroptosis, a mode of cell death resulting from iron-dependent lipid peroxidation and the accumulation of reactive oxygen radicals, was first introduced by Dr. Brent R. Stockwell in 2012^49^. Iron overload in cells can occur through endogenous or exogenous pathways^50^. The endogenous pathway is activated mainly by blocking the expression of intracellular antioxidant enzymes^51^, while the exogenous pathway is initiated mainly through the regulation of transport proteins such as lacto-transferrin (LTF)^52^ and transferrin (TF)^53^. In the past decade, research on iron death has increased, yet a complete comprehension of the concept remains elusive. Zinc, a non-redox-active metal, has been linked to neurodegeneration, particularly in AD, where it has been identified as a significant element in amyloid plaques^54^. To date, the precise process by which zinc mediates cell death remains elusive. Nonetheless, numerous hypothesized mechanisms pertaining to zinc's role in cell death converge on a shared position: zinc depletion stimulates the activation of caspases, ultimately resulting in apoptotic cell death. Thus, zinc exhibits a cytoprotective, rather than harmful, function. A newly published study^34^ showed that copper could induce cell death by targeting lipidated tricarboxylic acid (TCA) cycle proteins. This new mode of death is called ‘copper death’. Thus, whether copper metabolism can be linked to the pathogenesis of AD through TCA degeneration and how the copper-induced cell death mechanism plays a role in AD may require further study.

Additionally, we identified five hub genes. Among these, FGF2 encodes neurogenic factors for the proliferation and differentiation of pluripotent neural progenitor cells isolated from the brains of adult mice^55^. In the AD transgenic mouse model (APP + PS1 and J20), FGF2 gene expression mediated by adeno-associated virus serotype 2/1 hybridization (AAV2/1) significantly restored spatial learning, long-term hippocampal CA1 enhancement (LTP), and neurogenesis of the SGZ^56^. Interestingly, besides its neurogenic properties, FGF2 appeared to have anti-inflammatory and amyloid-reducing effects: AAV2/1-FGF2 mice injected with APP + PS1 showed decreased total a β and plaque burden and increased microglial proliferation around the plaque area. Moreover, treatment of primary cultured microglia with FGF2 enhanced the phagocytosis of Aβ, and infection of primary cultured neurons with AAV2/1-FGF2 reduced the production of Aβ, indicating that FGF2 not only had an effective effect on neurons but also had effective phagocytosis of microglia^56^. Therefore, FGF2 may be an effective agent for reducing AD lesions. Additional clinical experiments are needed to support this conclusion.

Beta-2-microglobulin encodes a serum protein associated with the major histocompatibility complex class I heavy chain on the surface of almost all nucleated cells. This protein has a predominantly pleated sheet structure that permits the formation of amyloid fibrils under certain pathological conditions. Recently, Professor Xin Wang's team published^57^ that β2M in peripheral blood can cross the blood–brain barrier into the CNS and impair neuronal synaptic function by inhibiting NMDA receptors, which may underlie cases of several cognitive disorders, but it is not clear whether β2M is deposited in the CNS. Subsequently, the team found that β2M levels were significantly upregulated in the brains of AD patients and bound to Aβ to form a β2M-Aβ copolymer, exacerbating its neurotoxicity, while knockdown of β2M almost antagonised the neurotoxicity of Aβ^58^. Therefore, targeting β2M could be a potential strategy for AD treatment.

The inhibition of glucose utilisation in the brain is associated with cognitive dysfunction in AD^59^. PTPRC is primarily associated with glucose metabolism in the brain. Protein Tyrosine Phosphatase Receptor Type C (PTPRC) encodes a protein that is a member of the protein tyrosine phosphatase (PTP) family. PTPs are known to directly affect cellular metabolism, and the expression of glycolytic enzymes is affected by various PTPs. For example, Cdc25A is a positive regulator of PKM2, which catalyzes the conversion of phosphoenolpyruvate to pyruvate during glycolysis.

Secreted phosphoprotein 1 (SPP1) and CD44 are closely associated with AD. Studies have shown that in a mouse model of AD, SPP1 is upregulated at the onset of synaptic phagocytosis in microglia and regulates the perivascular-microglia interaction network^60^. Thus, spp1 is required for activation of the complement initiator C1q and microglia phagocytosis of synapses, and deletion of Spp1 expression prevents synapse loss. Similarly, CD44 plays an important role in the development of AD. Exposure to Aβ_1-42_ leads to upregulation of CD44 variant genes, while knockdown of CD44 isoforms reduces neuronal apoptosis and acts as a neuroprotective agent. Thus, inhibition of CD44 activity may provide a new therapeutic approach for drug discovery^61^.

Using hub genes, we identified 6 of the 28 drugs (adalimumab, etanercept, infliximab, estradiol, prednisone, and progesterone) for which trials have been conducted to investigate their effects on AD, but no corresponding findings have been reported. The remaining 22 drugs were not found to be related to AD treatment, making them potential targets for AD.

In summary, our study has further revealed the existence of a strong correlation between the pathogenesis of AD and copper metabolism, but the specific mechanism of copper in this process requires further investigation. We identified five hub genes and established a series of molecular network enrichment pathways related to their functions. Through this initial exploration, we hope to uncover the “new veil” of the pathological process of AD, find new therapeutic targets for patients with AD, and improve the condition and alleviate pain. However, there are still limitations to our study. First, our results were not validated experimentally to demonstrated that genetic differences do exist. Secondly, we do not know exactly how the genes altered in the brains of AD patients are "linked" to the cu-metabolism genes. Thirdly, the safety and efficacy of drugs developed for Aβ must be established through animal studies and clinical trials. In conclusion, there is still much room for exploration in AD research.

## Data Availability

Publicly available datasets were analyzed in this study. These data can be found in the GEO data repository (https://www.ncbi.nlm.nih.gov/geo/) and include the accession number: GSE5281.
